# Are voltage-gated sodium channels on the dorsal root ganglion involved in the development of neuropathic pain?

**DOI:** 10.1186/1744-8069-7-16

**Published:** 2011-02-23

**Authors:** Wei Wang, Jianguo Gu, Yun-Qing Li, Yuan-Xiang Tao

**Affiliations:** 1 Department of Anesthesiology and Critical Care Medicine, Johns Hopkins University School of Medicine, Baltimore, Maryland 21205, USA; 2 Department of Anatomy, Histology and Embryology, K. K. Leung Brain Research Centre, the Fourth Military Medical University, Xi'an, 710032, China; 3 Department of Anesthesiology, University of Cincinnati College of Medicine, PO Box 670531, 231 Albert Sabin Way, Cincinnati, OH 45267-0531, USA

## Abstract

Neuropathic pain is a common clinical condition. Current treatments are often inadequate, ineffective, or produce potentially severe adverse effects. Understanding the mechanisms that underlie the development and maintenance of neuropathic pain will be helpful in identifying new therapeutic targets and developing effective strategies for the prevention and/or treatment of this disorder. The genesis of neuropathic pain is reliant, at least in part, on abnormal spontaneous activity within sensory neurons. Therefore, voltage-gated sodium channels, which are essential for the generation and conduction of action potentials, are potential targets for treating neuropathic pain. However, preclinical studies have shown unexpected results because most pain-associated voltage-gated channels in the dorsal root ganglion are down-regulated after peripheral nerve injury. The role of dorsal root ganglion voltage-gated channels in neuropathic pain is still unclear. In this report, we describe the expression and distribution of voltage-gated sodium channels in the dorsal root ganglion. We also review evidence regarding changes in their expression under neuropathic pain conditions and their roles in behavioral responses in a variety of neuropathic pain models. We finally discuss their potential involvement in neuropathic pain.

## Introduction

Neuropathic pain is a chronic condition that affects millions of people worldwide. It is characterized by pain hypersensitivity, including spontaneous ongoing or intermittent burning pain, an exaggerated response to painful stimuli, and pain in response to normally innocuous stimuli. Because the mechanisms of neuropathic pain induction and maintenance are far more complicated than previously assumed, current treatments can be ineffective or produce potentially severe adverse effects. Understanding molecular mechanisms of this disorder may allow improvement of its treatment.

**Figure 1:**
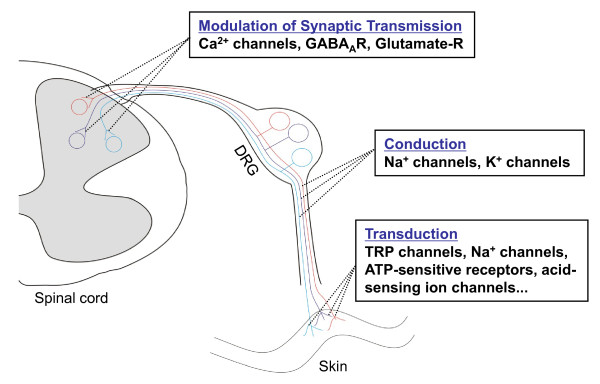
**Involvement of dorsal root ganglion (DRG) channels and receptors in the induction and modulation of pain**. A variety of DRG channels and receptors are involved in the transduction of noxious stimuli into electric impulses at the peripheral terminals of DRG neurons [e.g., transient receptor potential (TRP) channels, voltage-sensitive sodium (Na^+^) channels, ATP-sensitive receptors, acid sensing ion channels], in the conduction of action potentials along the axons [e.g., voltage-sensitive Na^+ ^channels and potassium (K^+^) channels], and in the modulation of neurotransmitter release at presynaptic terminals of primary afferents in the dorsal horn [e.g., voltage-gated calcium (Ca^2+^) channels, GABA receptors, and glutamate receptors].

It is generally believed that neuropathic pain is caused by changes in expression and function of receptors, enzymes, and voltage-dependent ion channels in peripheral nerves and dorsal root ganglion (DRG) neurons, as well as at synapses in the nociceptive pathway in the central nervous system [1,2]. DRG neurons express many kinds of ion channels/receptors. These channels and receptors have at least three functions (Figure 1): 1) Transduction (e.g., transient receptor potential channels, sodium channels, acid-sensing ion channels, and ATP-sensitive receptors that are expressed in the peripheral terminals of DRG neurons transduce noxious stimuli into electric impulses), 2) Conduction (e.g., sodium and potassium channels are involved in the propagation of action potentials), and 3) Modulation of synaptic transmission (e.g., voltage-gated calcium channels and glutamate receptors that are expressed on presynaptic terminals of the primary afferents in dorsal horn regulate the release of neurotransmitters). After nerve injury, injured and uninjured DRG neurons become more excitable and exhibit ectopic firing [3,4]. It is reasonable to conclude that this abnormal spontaneous activity might be related to nerve injury-induced changes in the density, distribution, and functional activities of voltage-gated sodium channels in the DRG neurons.

To date, at least nine subtypes of sodium channel have been cloned and identified on mammalian cells. All sodium channels consist of a central α-subunit and two auxiliary β-subunits. Nine α-subunits (Nav1.1-Nav1.9, also referred to as channels) and four β-subunits have been identified in mammals. The pore-forming α-subunit determines the primary function of sodium channels, but the kinetics and voltage-dependence of channel gating are in part modified by the β-subunits. The α-subunits form four homologous domains (I-IV), each of which contains six transmembrane α helices (S1-S6) and an additional pore loop located between the S5 and S6 segments. Voltage sensors of sodium channels are located in the highly conserved S4 transmembrane segments. Membrane depolarization produces changes in the transmembrane electric field and causes the S4 segment to spiral outward. This conformational change opens the pore. Following activation, sodium channels quickly inactivate to prevent further ion flow through the pore and to allow repetitive action potential firing of cells. Most voltage-gated sodium channels can be blocked by nanomolar concentrations of tetrodotoxin (TTX) and thereby are termed TTX-sensitive channels. These TTX-sensitive channels show rapidly activating and inactivating sodium currents. In contrast, Nav1.5, Nav1.8, and Nav1.9 are relatively resistant to this toxin and show sodium currents that are TTX-resistant [5].

**Figure 2:**
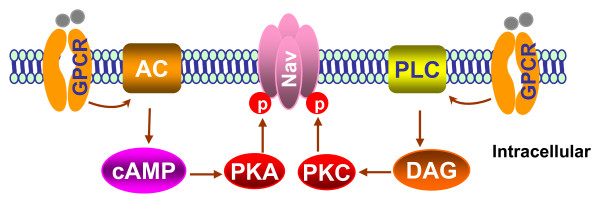
**Schematic representation of signaling pathways that modulate Na**^**+ **^**channels (Nav)**. The activation of G-protein-coupled receptors (GPCR) by their ligands activates adenylyl cyclase (AC) and phospholipase C (PLC), which produce cyclic adenosine monophosphate (cAMP) and diacylglycerol (DAG), respectively. cAMP then activates cAMP-dependent protein kinase (PKA), whereas DAG activates protein kinase C (PKC). Both PKA and PKC phosphorylate (P) the Na^+ ^channel to regulate its function.

**Figure 3:**
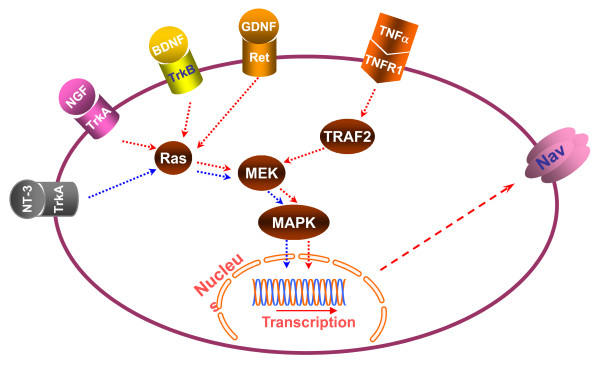
**Potential mechanisms by which sodium channel (Nav) expression is regulated**. Neurotrophins such as nerve growth factor (NGF), brain-derived neurotrophic factor (BDNF), and glial-derived neurotrophic factor (GDNF) bind to their respective receptors: tyrosine kinase receptor (Trk) A, TrkB, and Ret; receptor stimulation then activates the Ras/MEK/MAPK pathway. Activated MAPK promotes expression of sodium channels at the levels of mRNA and protein through unknown mechanisms (indicated by the red dashed arrows). An inflammatory cytokine, tumor necrosis factor α (TNFα), also up-regulates expression of sodium channels through activation of the TRAF2/MEK/MAPK pathway. In contrast, neurotrophin-3 (NT-3) down-regulates the expression of sodium channels through TrkA-mediated inhibition of the Ras/MEK/MAPK pathway (indicated by the blue dashed arrows). MAPK: mitogen-activated protein kinases; MEK: MAPK kinase; TNFR1: tumor necrosis factor receptor 1; TRAF2: TNF receptor-associated factor 2.

Voltage-gated sodium channels can be modulated by receptors coupled to intracellular signaling molecules (Figure 2). The modulation can occur through phosphorylation of specific residues on the α-subunit after the activation of cytoplasmic protein kinases. Two protein kinases, protein kinase A and protein kinase C, have been shown to target voltage-gated sodium channels. Both are activated by G-protein-coupled second messenger systems. The specific amino acid residues that are phosphorylated by these two kinases are located primarily on the linker between domains 1 and 2. The phosphorylation of voltage-gated sodium channels alters their function [6]. Moreover, the expression of voltage-gated sodium channels can be up-regulated by neurotrophins, including nerve growth factor, brain-derived neurotrophic factor (BDNF), and glial-derived neurotrophic factor (GDNF) (Figure 3) [7-9]. Interestingly, intrathecal injection of neurotrophin-3 causes significant decreases in the levels of Nav1.8 and Nav1.9 in L5 DRGs ipsilateral and contralateral to chronic constriction injury (CCI) of sciatic nerve [10]. In addition, inflammatory cytokines such as tumor necrosis factor α (TNFα) up-regulate the expression of Nav1.3, Nav1.8, and Nav1.9 and increase both TTX-sensitive and -resistant currents in the DRG neurons [11,12]. These effects of neurotrophins and pronociceptive cytokines on sodium channel expression might be mediated through regulation of intracellular downstream signaling pathways of their receptors, including p38 and ERK1/2 mitogen-activated protein kinase (Figure 3) [11,13].

Most sodium channels (except for Nav1.4, which is predominantly expressed in adult skeletal muscle [14] and Nav1.5, which is expressed in cardiac tissue) have been identified in adult DRGs [15]. Their expression level and the cell types to which they are localized in the DRG are distinct under normal conditions. Unexpectedly, preclinical studies indicate that peripheral nerve injury down-regulates most pain-associated voltage-gated channels in the injured DRG. Whether and how voltage-gated channels participate in nerve injury-evoked ectopic firing in the DRG neurons is still not unclear. In this review, we describe the expression and distribution of each sodium channel subtype in the DRG. We also review evidence regarding changes that occur in channel expression under neuropathic pain conditions and their roles in behavioral responses in a variety of neuropathic pain models. Finally, we discuss their potential involvement in this disorder.

### Nav1.1

Nav1.1 is a TTX-sensitive sodium channel [16,17], but the current properties of Nav1.1 have not been characterized in DRG neurons. In situ hybridization histochemistry has shown that Nav1.1 mRNA expression in DRGs is high in large-diameter neurons, moderate in medium-diameter neurons, and low in small-diameter neurons [18,19]. Approximately 25-33% of DRG neurons in naïve rats are positive for Nav1.1 mRNA [20]. Double immunostaining has shown that most Nav1.1-labeled cells are positive for NF200 (a marker for myelinated A-fibers), and that 79.2% of NF200-positive neurons express Nav1.1 mRNA. Research also has shown that 65.0% of Nav1.1-positive cells co-express neurotrophin-3 receptor tyrosine kinase C (TrkC; a marker for non-nociceptive mechanosensors) and that 51.6% of TrkC-labeled DRG cells are positive for Nav1.1 [21]. These findings indicate that Nav1.1 is expressed predominantly in the large-diameter A-fiber DRG neurons and that it might participate mainly in proprioceptive transmission (Table 1). It should be noted that approximately 11% of Nav1.1 mRNA-positive DRG neurons are positive for IB4 (a marker for small non-peptidergic nociceptive neurons), suggesting that Nav1.1 in these small-diameter DRG neurons may participate in nociceptive transmission and modulation [20]. Indeed, mutations in SCN1A (the gene for Nav1.1) have been associated with inherited epileptic syndromes [22] and familial hemiplegic migraine in humans [23]. Interestingly, preclinical studies showed that the level of Nav1.1 mRNA was decreased in the injured DRG after peripheral spinal nerve ligation (SNL) or spared nerve injury (SNI) [24,25]. Thus, whether and how DRG Nav1.1 is involved in neuropathic pain development is still elusive and remains to be further studied.

**Table 1:** Summary of sodium channel distribution and potential involvement in pain conditions

Channel	Distribution in normal DRG	Inflammatory pain		Neuropathic pain		Effect of manipulation on behavioral consequences		Human disorders
		mRNA	Protein	mRNA	Protein	Antisense/siRNA	Knock out	
1.1	High in large cells, low in small cells	Unchanged (carrageenan)	Unchanged (carrageenan)	Decreased (SNI, SNL)	N/A	N/A	N/A	Migraine, epilepsy
1.2	Very low in most conditions	Unchanged (carrageenan)	Unchanged (carrageenan)	Decreased (SNI, SNL)	N/A	N/A	N/A	Epilepsy
1.3	Extremely low in adult DRG	Increased (carrageenan)	Increased (carrageenan)	Increased (SNI, SNL)	Increased (SNI, SNL)	Effective on SCI and CCI, but no effect on SNI	No effect on acute, inflammatory, or neuropathic pain	Accumulates in neuromas of human painful neuropathy
1.6	High in large cells, low in small cells	Unchanged (carrageenan)	Unchanged (carrageenan)	Decreased (SNI, SNL)	N/A	N/A	N/A	N/A
1.7	Predominantly in small cells	Increased (carrageenan, CFA)	Increased (carrageenan, CFA)	Decreased (SNI, SNL)	Decreased (SNA, SNI, and SNL)	Effective on CFA	Effective on acute and inflammatory pain; no effect on neuropathic pain	Decreased in human injured DRG; accumulates in neuromas; mutations: PE, PEPD, and CIP
1.8	Exclusively in small cells	Increased (carrageenan)	Increased (carrageenan)	Decreased (SNI, SNL)	Decreased in L5 DRG (SNL, SNI) but increased in L4 DRG and sciatic nerve	Effective on CFA, SNL, and CCI	Effective on inflammatory pain; no effect on neuropathic pain	Accumulates in neuromas of human painful neuropathy
1.9	Selectively expressed in small cells	Increased (CFA), unchanged (carrageenan)	Unchanged (carrageenan)	Decreased (SNA, SNI, and SNL)	Decreased (SNA, SNI, and SNL)	No effect on SNL	Effective on inflammatory pain; no effect on neuropathic pain	N/A

Abbreviations: CCI, chronic constrictive injury; CFA, complete Freund's adjuvant; CIP, channelopathy-associated insensitivity to pain; DRG, dorsal root ganglion; N/A, not applicable; PE, primary erythermalgia; PEPD, paroxysmal extreme pain disorder; SCI, spinal cord injury; SNA, sciatic nerve axotomy; SNI, spared nerve injury; SNL, spinal nerve ligation.

### Nav1.2

Nav1.2 is one of the predominant sodium channels in the central nervous system; it is localized on dendrites, unmyelinated axons, and premyelinated axons [26]. The level of Nav1.2 mRNA expression in the adult DRG is very low [18], although its expression is moderate in early developmental stages. Peripheral nerve injury and inflammation do not alter the levels of Nav1.2 mRNA or protein in the DRG [19,24,25]. The evidence suggests that DRG Nav1.2 is unlikely to be involved in the development of neuropathic pain (Table 1).

### Nav1.3

Although Nav1.3 is expressed abundantly in DRG neurons during fetal and neonatal periods, it is normally undetectable in adult naïve DRG neurons [27]. However, it can be up-regulated in the injured DRG and ipsilateral dorsal horn after peripheral nerve injury. Approximately 37.5% of DRG neurons are Nav1.3-positive in the L5 DRG after sciatic nerve lesion and 15.8% after sural axotomy [28]. In situ hybridization histochemistry showed that, after L5 SNL, 40.7-47.2% of DRG neurons were Nav1.3 mRNA-positive cells, most of which were medium or large in size [20]. A recent study indicated that L5 ventral root transection produces a TNFα-dependent increase in Nav1.3 at both the mRNA and protein levels in the L4 and L5 DRGs [12]. Nav1.3 protein was also found to accumulate in neuromas of patients with painful neuropathy [29] and to up-regulate in second-order dorsal horn neurons after CCI [30]. These findings suggest that an increase in Nav1.3 in DRG and dorsal horn might be involved in nerve injury-induced pain hypersensitivities.

Despite the accrued evidence, the role of Nav1.3 in neuropathic pain behavior is still controversial. Hains et al. [31] reported that knockdown of DRG Nav1.3 via intrathecal administration of Nav1.3 antisense oligodeoxynucleotides (ASO) attenuated pain hypersensitivities induced by spinal cord injury and sciatic nerve CCI. In contrast, Lindia et al. [28] found that intrathecal administration of Nav1.3 ASO did not attenuate SNI-induced mechanical or cold allodynia, although it did significantly block the SNI-induced increase in DRG Nav1.3. In addition, neuropathic pain development remained intact in both conventional and conditional Nav1.3 knockout mice [32]. Furthermore, ectopic discharges from the injured nerves were unaffected in the absence of Nav1.3 in conventional knockout mice [32]. These results suggest that Nav1.3 is unlikely to be a key player in the induction of abnormal spontaneous activity in injured neurons (Table 1).

### Nav1.6

Nav1.6 is predominantly located in the Nodes of Ranvier of both motor and sensory axons in the peripheral and central nervous systems [33]. In adult DRG, the cellular distribution pattern of Nav1.6 is similar to that of Nav1.1. That is, it is highly colocalized with NF200 [20], indicating that Nav1.6 is an A-fiber-specific channel (Table 1).

Nerve injury alters expression of DRG Nav1.6. Its mRNA is down-regulated in the injured L5 DRG following SNL and SNI [25]. However, in a rat model of infraorbital nerve injury, the level of Nav1.6 protein was found to be significantly increased proximal to the lesion site [34], suggesting that it might be transported quickly to the peripheral terminals under neuropathic pain conditions. Whether this increase participates in the generation of abnormal spontaneous activity in the injured DRG neurons remains to be further studied.

### Nav1.7

Nav1.7 is widely expressed in sensory, sympathetic, and myenteric neurons [18,35,36]. In the DRG, Nav1.7 is distributed predominantly in small-diameter neurons [18,19]. Double-labeling studies have shown that most NF200-negative neurons (>99%) express Nav1.7 mRNA [20] (Table 1). Nav1.7, as well as Nav1.6, Nav1.8, and Nav1.9, is present in most intra-epidermal free nerve endings [37], suggesting that these sodium channels are poised to participate in amplification of generator potentials, and sets the gain on nociceptors. Nav1.7 displays slow closed-state inactivation [38]. As a result of this characteristic, Nav1.7 is unable to respond during high-frequency stimulation, but it responds to small depolarizing stimuli close to the resting membrane potential [38]. Nav1.7 may be physiologically coupled to Nav1.8 within DRG neurons. It serves to boost subthreshold stimuli, resulting in the activation of Nav1.8, which recovers rapidly from inactivation and produces high-frequency action potentials [39]. The evidence indicates that Nav1.7 is expressed mainly on C- and Aδ-nociceptive fibers, contributes to amplification of generator potentials, and sets the gain on nociceptors [40,41]. Indeed, data from animal studies have indicated that Nav1.7 plays a crucial role in nociception. Nav1.7 mRNA and protein are up-regulated in DRG after peripheral inflammation induced by carrageenan or complete Freund's adjuvant (CFA) [19,42]. In addition, knockdown of DRG Nav1.7 significantly prevents the development of hyperalgesia in response to CFA [43]. Nav1.7 knockout mice also fail to develop hyperalgesia in several inflammatory pain models (Table 1) [44].

In humans, mutations in the SCN9A gene (which encodes Nav1.7) are associated with three known pain disorders: channelopathy-associated insensitivity to pain (CIP), paroxysmal extreme pain disorder (PEPD), and primary erythermalgia (PE) [45,46]. Patients with CIP lose normal response to painful insults such as puncture wounds, bone fracture, biting, or contact with hot surfaces, although other sensory responses are normal [47]. PEPD is characterized by severe burning pain in the rectal, ocular, and submandibular regions, and PE by burning pain and redness of the extremities [48]. The evidence indicates that DRG Nav1.7 plays a key role in acute and inflammatory pain.

In contrast to its role in acute and inflammatory pain, whether Nav1.7 is involved in nerve injury-induced neuropathic pain is still unclear. Nav1.7 protein and current are both increased in the DRG in a rat model of painful diabetic neuropathy [49,50], whereas the amount of Nav1.7 protein is reduced in the injured DRG after SNL, SNI, and sciatic nerve axotomy in animals [25,51]. The level of Nav1.7 protein is also decreased in the injured DRG of humans after peripheral axotomy or traumatic central axotomy [52], but Nav1.7 protein has been observed to accumulate in painful neuromas of amputees with phantom limb pain [29,53]. Interestingly, a mouse behavioral study showed that conditional knockout of DRG Nav1.7 did not affect SNL-induced development of mechanical allodynia [54]. Thus, it remains questionable whether DRG Nav1.7 has a role in the development of neuropathic pain.

### Nav1.8

Nav1.8 is a sensory neuron-specific voltage-gated sodium channel that is expressed exclusively in small-diameter nociceptive DRG neurons [55]. Double-labeling studies have shown that 60.0% of Nav1.8-positive DRG neurons are IB4-positive [20]. Nav1.8 mRNA and protein are increased in DRG neurons of rodents following injection of carrageenan into a hind paw [19,56,57]. Knockdown of DRG Nav1.8 reduces the mechanical allodynia caused by intraplantar injection of CFA [58]. Furthermore, Nav1.8 knockout mice display impaired thermal and mechanical pain hypersensitivity in response to carrageenan-induced inflammation [59]. These results indicate that Nav1.8 in DRG plays a key role in inflammatory pain (Table 1).

In contrast to inflammatory insult, peripheral nerve injury down-regulates Nav1.8 mRNA and protein expression in the small-diameter neurons of the injured DRG [25,60-62]. This down-regulation might be related to epigenetic gene silencing. Peripheral nerve injury up-regulates neuron-restrictive silence factor (NRSF) expression in the DRG and promotes NRSF binding to the neuron-restrictive silencer element within the Nav1.8 gene, thereby silencing its expression [63]. Interestingly, an increase in Nav1.8 protein was observed in the large-diameter neurons of the uninjured L4 DRG after L5 SNL [25,64]. After L5 SNL, Nav1.8 immunoreactivity was also strikingly increased in the uninjured C-fibers of sciatic nerves [62]. Moreover, intrathecal administration of Nav1.8 ASO prevented the nerve injury-induced increase in Nav1.8 in the sciatic nerve [62]. TNFα might participate in this increase because inhibition of TNFα synthesis and knockout of TNFα strongly inhibited nerve injury-induced up-regulation of DRG Nav1.8 [12]. In patients with chronic neuropathic pain, Nav1.8 channel expression was reported to be increased in the nerves proximal to injury sites [29]. These results suggest that peripheral nerve injury might trigger TNFα-dependent translation of Nav1.8 in uninjured DRG neurons and promote the transportation of Nav1.8 from the uninjured DRG cell bodies to their axons.

The elevated Nav1.8 in uninjured DRG neurons and their axons might account, at least in part, for the abnormal spontaneous activity and behavioral tactile allodynia observed after nerve injury. Behavioral studies appear to support this conclusion. Intrathecal administration of Nav1.8 ASO attenuated nerve injury-induced mechanical and thermal hyperalgesia [62], although it failed to reduce mechanical allodynia in vincristine-induced neuropathic pain [58]. Small interfering RNAs that specifically target Nav1.8 were able to reverse mechanical allodynia in a rat CCI model when administered intrathecally [65]. Additionally, a Nav1.8 blocker, A-803467, dose-dependently attenuated mechanical allodynia in rat neuropathic pain models of SNL and sciatic nerve injury [66]. Interestingly, neuropathic pain develops normally in the Nav1.8 knockout mouse [59,67]. Moreover, the use of diphtheria toxin to selectively delete most nociceptors (> 85%) that predominantly express Nav1.8 (as well as Nav1.7 and Nav1.9) in mouse DRG did not affect nerve injury-induced mechanical or thermal pain hypersensitivities [68]. These conflicting results indicate that the role of DRG Nav1.8 in neuropathic pain development is still uncertain and needs to be investigated further.

### Nav1.9

Nav1.9 is selectively expressed in small-diameter (<30 μm) DRG neurons. Sixty-two percent of Nav1.9-positive DRG neurons are IB4-positive [20]. DRG Nav1.9 is also highly co-localized with TRPV1, purinergic P2X3 receptor, and B2 bradykinin receptor [69]. Although carrageenan injection does not alter the expression of Nav1.9 mRNA or protein in DRG [19], the level of Nav1.9 mRNA in DRG neurons is significantly increased in the CFA model [70]. Nav1.9 knockout mice exhibit blunted pain behaviors in response to formalin, carrageenan, CFA, and prostaglandin E2 [71]. Similar to Nav1.7 and Nav1.8, DRG Nav1.9 may be required for the development of inflammatory pain (Table 1).

In contrast to its involvement in inflammatory pain, DRG Nav1.9 might not contribute to the development of neuropathic pain. The levels of Nav1.9 mRNA and protein, as well as its current density, are reduced in the DRG after sciatic nerve axotomy [60,72], SNL, and SNI [25,61]. In addition, intrathecal administration of Nav1.9 ASO has no effect on SNL-induced neuropathic pain [64]. Intact mechanical and thermal pain hypersensitivities were observed in Nav1.9 knockout mice after SNI and partial ligation of the sciatic nerve [69,71]. Current preclinical evidence does not support a role for DRG Nav1.9 in the development of neuropathic pain.

## Conclusion

Voltage-gated sodium channels conduct sodium ion influx and control action potential generation. It has been assumed that DRG voltage-gated sodium channels participate in induction of neuropathic pain. However, as summarized in Table 1, most voltage-gated sodium channels in DRG (with the exception of Nav1.3) are down-regulated after peripheral nerve injury. This down regulation is in contrast to the increased expression that is observed under persistent inflammatory pain conditions. The mechanisms that underlie the expression changes in neuropathic pain are still unclear. As discussed above, neurotrophins (e.g., BDNF and GDNF) and cytokines modulate voltage-gated sodium channel expression (Figure 3). Up-regulation of the neurotrophic factors and the release of cytokines cannot explain the down-regulation of voltage-gated sodium channels in the DRG under neuropathic pain conditions [73,74]. More importantly, most behavioral findings from animal models do not support a role for DRG voltage-gated sodium channels in neuropathic pain (Table 1). Interestingly, the use of sodium channel blockers (such as lidocaine) in patients can effectively inhibit a variety of neuropathic pain syndromes [75], although they also produce significant side effects. Inconsistent results between clinical and laboratory observations necessitate careful consideration of the differences between human and animal models and the methods for pain assessment. Therefore, a possible role for DRG voltage-gated sodium channel function in neuropathic pain cannot be excluded and remains to be further investigated.

## Competing interests

The authors declare that they have no competing interests.

## Authors' contributions

WW and YXT participated in the drafted manuscript. JG, YQL, and YXT contributed to critical review of the manuscript. All authors have read and approved the final manuscript.
